# Neurotrophins and Matrix Metalloproteinases in Treatment-Resistant Schizophrenia: Effects of Electroconvulsive Therapy on Serum Biomarkers and Clinical Outcomes: A Preliminary Study

**DOI:** 10.3390/biomedicines14071535

**Published:** 2026-07-08

**Authors:** Anna Maria Szota, Małgorzata Ćwiklińska-Jurkowska, Izabela Radajewska, Kinga Lis, Przemysław Grudzka, Wiktor Dróżdż

**Affiliations:** 1 Department of Psychiatry, Ludwig Rydygier Collegium Medicum in Bydgoszcz, Nicolaus Copernicus University in Toruń, 9 Curie-Skłodowskiej Street, 85-094 Bydgoszcz, Poland; przemyslaw.grudzka@cm.umk.pl (P.G.); wikdr@cm.umk.pl (W.D.); 2 Department of Biostatistics and Biomedical Systems Theory, Ludwig Rydygier Collegium Medicum in Bydgoszcz, Nicolaus Copernicus University in Toruń, 13-15 Jagiellonska Street, 85-067 Bydgoszcz, Poland; mjurkowska@cm.umk.pl; 3 Medical Center Gizińscy, 9 Leśna Street, 85-676 Bydgoszcz, Poland; izabela.radajewska@gizinscy.pl; 4 Department of Allergology, Clinical Immunology and Internal Diseases, Ludwig Rydygier Collegium Medicum in Bydgoszcz, Nicolaus Copernicus University in Toruń, 75 Ujejskiego Street, 85-168 Bydgoszcz, Poland; kinga.lis@cm.umk.pl

**Keywords:** electroconvulsive therapy (ECT), treatment-resistant schizophrenia (TRS), BDNF, metalloproteinases, TIMP-1

## Abstract

**Background:** Available data indicate that the development of refractory schizophrenia may result from neuroinflammation, dysregulation of neurotrophins and metalloproteinases (MMPs), oxidative stress (OS), and hormonal imbalance. Electroconvulsive therapy (ECT) is an effective therapeutic option for drug resistance, but its impact on brain-derived neurotrophic factor (BDNF) and MMPs remains underinvestigated. This study evaluates the influence of ECT on serum BDNF, MMPs (MMP-7, MMP-9, MMP-14), and tissue inhibitors of metalloproteinases (TIMP-1, TIMP-2, TIMP-3) in patients with treatment-resistant schizophrenia (TRS). Another goal of this study is an assessment of the relationships between these biomarkers and the intensity of schizophrenia symptoms. **Methods:** Serum concentrations of the aforementioned biomarkers were measured prior to and after ECT in eight patients and 13 healthy controls. The severity of schizophrenia symptoms was evaluated with the Positive and Negative Syndrome Scale (PANSS). **Results:** Bayesian analysis comparing pre-ECT serum concentrations of BDNF, MMPs, and their inhibitors in TRS patients with a control group showed a significant difference only for MMP-9. Furthermore, convincing evidence of a correlation between MMP-9 and MMP-14 was found in TRS patients. Although the ECT therapy did not result in changes in the serum concentrations of the studied biomarkers, substantial improvement in schizophrenia symptoms on the PANSS was observed. **Conclusions**: No significant biomarker changes (post- versus pre-treatment) were detected in this small exploratory cohort. Whether these biomarkers are involved in neuroinflammation (as possible contributors to the development of TRS) remains an open question, and therefore, further research on biomarkers in cerebrospinal fluid (CSF) may be suggested.

## 1. Introduction

Schizophrenia (SCZ) is a complex, severe, and lifelong mental disorder characterized by psychotic symptoms (delusions and hallucinations), cognitive dysfunction, mood changes, and anhedonia. Pharmacotherapy with antipsychotic medication is a cornerstone treatment of this disorder; however, approximately 20–50% of patients do not respond, or respond poorly [1,2,3]. Consequently, SCZ progresses to treatment-resistant schizophrenia (TRS) [4] or to ultra treatment-resistant schizophrenia (UTRS) if resistance to clozapine develops [5]. The management of drug resistance remains a challenge, and the most common approach is augmentation with additional antipsychotics, mood stabilizers, anxiolytics, and antidepressants [5,6,7]. Alternatively, non-pharmacological interventions for TRS patients include electroconvulsive therapy (ECT), acknowledged as an effective and well-tolerated treatment method, usually associated with mild adverse effects [5,6,7,8,9]. Other neurostimulation techniques such as repetitive transcranial magnetic stimulation (rTMS) or transcranial direct current stimulation (tDCS) can also be used; nevertheless, the evidence for their effectiveness in TRS remains inconclusive. Therefore, neither method has gained approval for widespread clinical use [10,11]. The detailed neurobiological mechanisms responsible for TRS remain elusive, but available data indicate that neuroinflammation, oxidative stress, hormonal axis imbalance, disturbances in neurotrophins, and dysregulation of dopamine, glutamate, and serotonin pathways in the brain may be associated with TRS development [1,12,13].

Data from previous studies suggest that deregulation of synaptic plasticity with downstream alterations of neurotrophins is an important process involved in SCZ etiopathology [14,15]. Brain-derived neurotrophic factor (BDNF) is the most widely distributed neurotrophin in the central nervous system (CNS) [16]. This protein plays an important role in brain development, neurogenesis, and neuronal growth [17]. BDNF regulates neuroplasticity of the brain and peripheral nervous system by improving the survival, growth, and differentiation of nerve cells and synapses. BDNF is also a crucial modulator of cognitive functions such as memory, learning, attention, and executive function, which are disturbed to a various extent in SCZ [18,19,20]. Additionally, decreased serum or plasma BDNF levels have been associated with positive and negative symptoms of SCZ [21,22]. BDNF is initially synthesized as a precursor protein, pre-pro-BDNF, and converted to mature BDNF by extracellular proteases such as plasmin and matrix metalloproteinase-9 (MMP-9) [19,23].

MMP-9 is one of the matrix metalloproteinases (MMPs), produced by resident cells, such as neurons, astrocytes, microglia, and by infiltrating cells, including granulocytes and macrophages. MMPs are involved in growth regulation, proliferation, and cell apoptosis [24]. MMP-9 is mainly responsible for the regulation of synaptic plasticity and perineuronal net formation and integrity; it also regulates the migration of neuroimmune and inflammatory cells and takes part in myelination [25]. Moreover, MMP-9 plays a pivotal role in the degradation and remodeling of the extracellular matrix and regulates inflammatory processes, thereby affecting cytokine and chemokine concentrations, which are altered in SCZ [24,26]. Concurrently, MMP-9 expression is regulated by cytokines, within a bidirectional regulatory loop [27]. MMP-9, expressed at low levels in most cell types, is highly responsive to stimulation and has proinflammatory properties [28]. Similar to BDNF, MMP-9 is produced in the form of an inactive pro-enzyme, and its activation and functional regulation are controlled by tissue inhibitor of metalloproteinases-1 (TIMP-1) [1,29]. Interestingly, upregulated blood levels of MMP-9 were found in schizophrenia spectrum disorders and have been related to disease severity [30]. The ratio of MMP-9/TIMP-1 was significantly different in patients with SCZ as compared with healthy controls [31]. Previous studies have also shown that MMP-9 impacts cognitive functions such as memory and learning by inducing late-phase long-term potentiation (L-LTP) that modulates hippocampal synaptic plasticity [32]. Simultaneously, TIMP-1 may block L-LTP. Therefore, the imbalance between MMP-9 and TIMP-1 may influence the regulation of LTP processes and synaptic plasticity, which are seriously impaired in SCZ [31,33]. Growing evidence indicates a link between oxidative stress (OS), MMP-9, neuroinflammation, and altered expression of both OS factors and MMP-9 in schizophrenia [34,35].

Tylec et al. (2021) [24] suggested that production of other MMPs, such as MMP-7 and MMP-10, might be increased in SCZ. Both MMP-7 and MMP-10 are secreted from microglia in response to TNF-α [36,37]. In addition, MMP-10 activates MMP-7 [38], which in turn activates MMP-2 [39]. Increased production of MMP-2 impairs the permeability of the blood–brain barrier (BBB), allowing peripheral cytokines to enter the brain, which may lead to the persistent brain inflammation observed in SCZ [36].

Few studies on neurotrophins and MMPs have been performed in TRS patients. Decreased serum BDNF concentrations before ECT in TRS patients compared with healthy controls have been observed [40,41,42,43]. Increased serum MMP-9 levels [1,19] or lack of difference in its concentration between TRS patients and the control group have been reported [27]. Additionally, no significant serum TIMP-1 and TIMP-2 concentration differences were found in TRS patients compared with controls [1,27]. The effectiveness of treatment with ECT plus antipsychotics in TRS has been confirmed in multiple studies [44,45,46]. This therapeutic effect, reflected in improvements in SCZ symptoms, may be linked to ECT’s effects on neurotrophins, MMPs, and their inhibitors. Evidence indicates that ECT may have an ambiguous effect on serum BDNF: some studies have reported significant increases in BDNF post-ECT [18,41,47], whereas others have found no changes [40,42,43,48,49]. In the case of serum TIMP-1 and TIMP-2, no alterations were found, whereas serum MMP-9 concentration decreased post-ECT [27]. So far, neither the involvement of MMP-7, MMP-14, and TIMP-3 in TRS development, nor the impact of ECT on these parameters in serum has been studied.

Considering the above findings, the assessment of serum levels of BDNF, MMP-7, MMP-9, MMP-14, TIMP-1, TIMP-2, and TIMP-3 in TRS patients was the primary goal of this exploratory study. Additionally, the effect of ECT on the concentrations of these biomarkers and their relationship with the clinical evaluation of schizophrenia symptoms were assessed.

## 2. Materials and Methods

### 2.1. Participants and Enrolment Criteria

The study participants comprised eight patients (5 men and 3 women), diagnosed with schizophrenia according to the ICD-10 criteria. The patients were recruited at the Psychiatry Clinic of the University Hospital No.1 in Bydgoszcz (Poland). The recruitment period lasted a year and a half but was prematurely terminated because of the COVID-19 pandemic. In addition, a significant number of patients did not consent to either ECT treatment or participation in this study, which affected the number of patients recruited. The mean age of the patients was 35 (20–41 years), and they had been suffering from schizophrenia for between 3 and 23 years (an average of 13.9 years). The participants had previously undergone at least two consecutive unsuccessful trials of antipsychotic treatment before diagnosis of treatment resistance was made. The majority of the patients (*n* = 6) suffered from paranoid SCZ, and two from residual SCZ. Among them, five patients met the criteria for UTRS, as no clinical improvement to clozapine treatment had been observed [50,51]. Consequently, UTRS patients were treated with higher doses of antipsychotic drugs expressed as chlorpromazine equivalent doses, compared with patients treated with other antipsychotic drugs [52]. Four patients took antipsychotics at doses equivalent to >1000 mg/day of chlorpromazine, calculated using the Antipsychotic Dose Conversion Calculator (https://psychopharmacopeia.com/antipsychotic_conversion.php; accessed on 10 June 2024). Past substance use disorder was diagnosed in five patients, two of whom had nicotine dependence only. A history of suicidal attempts was found in three patients. All drug-resistant patients agreed to ECT therapy, and written informed consent was obtained before the first ECT session. The severity of SCZ symptoms was evaluated by psychiatrists (I.R. and P.G.) using the PANSS, performed before the first ECT session and on the next day after the last ECT session. Thirteen healthy individuals (11 women and 2 men, mean age 33 years) with no history of past or current mental disorders constituted the control group.

Exclusion criteria for both TRS patients and the control group were serious neurological, cardiological, and autoimmune diseases, and confirmed current addiction to any psychoactive substances, including alcohol. Additional exclusions applied to patients with significant medical conditions such as uncontrolled diabetes mellitus or other metabolic disorders, decompensated renal failure, acute glaucoma attack, stroke within the last 4 weeks, severe lung disease, intracranial neoplasm, and retinal detachment. Moreover, patients who were unable to sign the informed consent for ECT were excluded from this study.

Written informed consent was obtained from all participants in this study, which was approved by the Bioethics Committee of the Nicolaus Copernicus University in Toruń, Collegium Medicum in Bydgoszcz, Poland (approval number KB 631/2018). The standards of this study, described in the Declaration of Helsinki, were respected by the researchers.

### 2.2. Protocol of Electroconvulsive Therapy

Before the first session of ECT, each TRS patient underwent a physical and neurological examination and had a computed tomography (CT) scan. Blood and urine tests were also performed. The effectiveness of ECT was evaluated as a change in the PANSS score (pre-ECT vs. post-ECT). Each patient had 3 sessions of ECT per week, conducted every second day, with the total number of sessions ranging from 11 to 15, with a mean of 13 ECT sessions. ECT was conducted under general anesthesia induced with i.v. thiopental (3–4 mg/kg, i.v.). Muscle relaxation was achieved using succinylcholine (0.5–1.5 mg/kg, i.v.) [46,53,54]. Electrodes were placed frontotemporally for bilateral stimulation. One adequate seizure of a high-amplitude, slow wave with postictal suppression, lasting more than 20 s, was induced by the Thymatron device (Thymatron System IV, Somatics; Lake Bluff, IL, USA). The stimulus charge was maintained unless brief seizures occurred, in which case the charge was increased by 50%. The total number of ECT sessions for each patient was established on the basis of clinical improvement and adverse events, which possibly developed during ECT.

### 2.3. Immunology Parameter Assays

Serum samples for BDNF, MMP-7, MMP-9, MMP-14, TIMP-1, TIMP-2, and TIMP-3 were collected in fasted patients (7.00–7.30 a.m.), from all participants in this study. In the TRS patients, blood collection was conducted twice (pre-ECT and after the last ECT session), but healthy individuals had only one blood collection on the screening day. The collected blood was centrifuged 45 min after collection (3000 rpm for 10 min). Aliquoted serum was separated into sterile 1.5 mL tubes and stored at −80 °C until assay. Immunological parameters were measured using ELISA kits (BDNF, kit number: SEA011Hu; MMP-7, kit number: SEA102Hu; MMP-9, kit number: SEA553Hu; MMP-14, kit number: SEC056Hu; TIMP-1, kit number: SEA552Hu; TIMP-2, kit number: PAA128Hu06; TIMP-3, kit number: SEA129Hu; Cloud-Clone Corp, Katy, TX, USA). The measured levels of the above parameters were expressed as pg/mL.

### 2.4. Statistical Analysis

To test the null hypotheses of equality of means parametrically, logarithmic transformations were applied. For testing differences between distributions of measures before and following ECT and for the control group, the Bayesian *t*-tests [55] were applied.

For the comparison of values between patients and control subjects, Bayes factor independent samples inference was employed. Bayes factor independent samples test was performed with the Rouder method, with the assumption of unequal variances between the control and pre-ECT groups. To calculate posterior distributions, the adaptive quadrature method with a tolerance level of 0.000001 and a maximum iteration number of 2000 were applied for estimates of the integral.

Diffuse a priori for unknown variance were used together with a prior on the mean conditional on variance.

To compare the pre-ECT with the post-ECT results, Bayes factor dependent sample tests were applied. The Bayesian-related sample criteria for the comparison between pre-ECT and post-ECT were a tolerance level of 0.000001 and a maximum iteration number of 2000 for the adaptive Gauss–Lobatto quadrature numerical method. A Jeffreys prior with a parameter of 4 was applied a priori for unknown unequal variances and a diffuse prior mean [55].

The significance of seven serum parameters was corrected for multiple testing using the false discovery rate [56]. A 0.05 level was set as the threshold for significance for multiple testing.

The values of the Bayes factor indicate how much the data support one hypothesis compared to another. Additionally, 95% credible intervals were provided [57].

The relationships between variables within groups were examined by Bayes factor inference for pairwise correlations between BDNF, MMPs, and inhibitor concentrations in TRS patients for both pre-ECT and post-ECT.

Calculations were performed with PS IMAGO PRO v. 9.0, based on the IBM SPSS Statistics v.29 analytical engine (IBM SPSS Statistics: https://en.predictivesolutions.pl/ps-imago-pro,en, accessed on 16 May 2025).

## 3. Results

Comparisons of serum BDNF, MMP-7, MMP-9, MMP-14, TIMP-1, TIMP-2, and TIMP-3 for pre-ECT versus control subjects showed that the only clear difference was found for MMP-9. The average value of MMP-9 in the control subjects was 255.7 pg/mL, whereas in the TRS patients, it was 68.2 pg/mL (Table 1a,b).

The calculated Bayes factor was for the null hypothesis H_0_ versus the alternative hypothesis H_1_, where H_0_ is the null hypothesis of no difference between the pre-ECT and control groups. The value of the Bayes factor for MMP-9_l:BF10 = 1/BF01 =1/0.001 = 1000, indicating that the data support the alternative hypothesis of a difference between groups 1000 times more than the null hypothesis (Table 1b). This is decisive (extremely strong) evidence for the alternative hypothesis of a difference between the control and pre-ECT. This is concordant with the Benjamini–Hochberg correction for multiple comparisons (Table 1b), controlling the false discovery rate (equal to 0.007 for MMP-9_l).

According to Jeffreys’ scale [58] of the Bayes factor, the data give support for H_1_. The BF for the remaining variables (BF01) indicated weak support for H_0_ for MMP-14_l, TIMP-2_l, and TIMP-3_l, with Bayes factors of 2.909 and 2.503, respectively (Table 1b).

For BDNF_l and TIMP-1_l (prior to ECT) Bayes factors of 3.221 and 3.196, respectively (Table 1b), suggest moderate support for H_0_.

Additionally, the 95% confidence intervals (Table 1c) confirm these results: only for the MMP-9 prior to ECT was the CI (−0.878 to −0.337) wholly on the left side of zero, whereas the 95% confidence intervals for the remaining variables contained zero.

Correlations between particular biomarkers are presented in Table 2 and Table S1. A significant positive correlation between MMP-14 and MMP-9 (r = 875; BF = 14.67; 95% CI = 0.424 to 0.971) was found, which means that the increase in MMP-14 increase is related to an increase in MM9 (Table 2 and Table S1). Moreover, a moderate negative correlation between MMP-14 and TIMP-2 was found (r = −747; BF = 2.6; 95% CI = −0.928 to −0.122), indicating that a lower concentration of the inhibitor TIMP-2 was related to higher MMP-14 values.

Correlations between particular biomarkers and the PANSS scores pre-ECT are presented in Table 3 and Table S2. Weak evidence (BF_10_ = 1.76; 95% confidence interval = 0.038 to 0.915) was found only for the positive correlation between PANSS positive symptoms and MMP-7 pre-ECT.

Concentration changes in BDNF, MMP-7, MMP-9, MMP-14, TIMP-1, TIMP-2, and TIMP-3 post-ECT versus pre-ECT are presented in Table 4a.

Bayesian related-samples *t*-tests for comparisons between pre-ECT and post-ECT are presented in Table 4b,c. For MMP-9, TIMP-2, and TIMP-3, values of Bayes factors over 3 indicate moderate support for the null hypothesis (H_0_) of no difference between pre- ECT and post- ECT.

For BDNF, MMP-14, MMP-7, and TIMP-1, values of H_0_ between 1 and 3 indicate weak support for the null hypothesis (H_0_) of no difference between pre-ECT and post-ECT. Next, probabilities a priori were calculated by prior on variance, according to Jeffreys 4 method, and diffuse prior on mean given variance. Resulting 95% confidence intervals were calculated (Table 4c), and all of them contained 0.

A weak positive correlation between MMP-14 and TIMP-1 after ECT (BF = 1.44; 95% confidence interval = 0.673 to 0.696) was found (Table 5a,b). This result indicates that increased serum levels of MMP-14 are associated with increased TIMP-1 concentration in TRS patients.

The only convincing support for a correlation between the severity of SCZ symptoms, evaluated with the PANSS, and the measured proteins was found for the MMP-14 concentration and the positive symptoms score (BF_10_ = 1/0.125 = 8.03; 95% confidence interval= 0.337 to 0.965) post-ECT (Table 6a,b).

## 4. Discussion

This study evaluated the possible involvement of BDNF, MMP-7, MMP-9, MMP-14, TIMP-1, TIMP-2, and TIMP-3 in treatment-resistant schizophrenia and the impact of ECT on these biomarkers. Moreover, correlations between BDNF, MMPs, their inhibitors, and the severity of SCZ symptoms were analyzed.

Our study shows that the concentrations of BDNF, MMP-9, TIMP-1, TIMP-2, and TIMP-3 in serum were decreased in TRS patients before ECT compared to the control group. This difference was not robust, except for MMP-9 (Table 1a). The calculated value of the Bayes factor for MMP-9 gave enormously strong evidence (>1000) for the existing difference between TRS patients and the control group. This result was also confirmed with the Benjamini–Hochberg FDR correction method (FDR = 0.006) (Table 1b) and calculated confidence intervals (95% CI = −0.878 to −0.337) (Table 1c). Serum concentrations of MMP-7 and MMP-14 were increased in TRS patients, but the differences did not reach statistical significance (Table 1a). Our findings on BDNF in TRS patients align with previous results published by other authors [40,41,42,43] and contradict the research of Yamamori et al. (2013) [19], who found no difference in serum BDNF in TRS patients versus the control group. The values of MMP-9 in our study are in inconsistent to those of other studies, where an increased concentration of MMP-9 has been reported [1,19]. Regarding the TIMP-1 and TIMP-2 levels, our results are in line with previous data, indicating no significant differences for both inhibitors in TRS patients compared to the control subjects [1,27]. Currently, evidence on the potential role of MMP-7, MMP-14, and TIMP-3 in TRS patients is lacking.

As previously mentioned, BDNF is synthesized as a precursor protein, pre-pro-BDNF, mainly in the brain, but also in the megakaryocytes, and converted to mature BDNF by MMP-9 [19,23,59]. The concentration of serum MMP-9 in our patients was significantly decreased, so the production of mature BDNF, at least in part, may be disrupted by an insufficient concentration of MMP-9. Furthermore, some data indicate that chronic antipsychotic use in patients suffering from SCZ may have a direct impact on both BDNF and MMP-9. However, the findings are inconsistent. Mikulic et al. (2025) [60] reported an increased serum concentration of BDNF in SCZ patients chronically treated with olanzapine or risperidone. Conversely, other studies do not confirm the influence of long-term antipsychotic treatment (haloperidol, olanzapine, risperidone, quetiapine, aripiprazole) on serum BDNF levels [61,62,63,64,65]. In the case of clozapine, which is approved for the treatment of drug-resistant schizophrenia, an increased concentration of BDNF [66,67], or decreased BDNF levels were found [14,67], especially in responders to this medication. Additionally, some research found that serum BDNF concentration may be positively correlated with the daily dose of clozapine and duration of the treatment [22,66,68], while other studies found no such association [19,60]. Moreover, many factors, i.e., stress associated with psychotic symptoms, duration of illness, type of SCZ, and depressive symptoms, may also be involved in serum BDNF reduction [69].

A decrease in BDNF concentration in patients with TRS may also be correlated with changes in the concentration of other neurotrophic factors, such as VEGF (vascular endothelial growth factor), GDNF (glial cell line-derived neurotrophic factor), NGF (nerve growth factor), and NT-3 (neurotrophin-3). These changes result from a complex interplay within a network of interconnections and interactions between the aforementioned factors. VEGF plays a crucial role in angiogenesis, neurodevelopment, and synaptic plasticity, exerting a neuroprotective effect. Furthermore, VEGF and BDNF enhance cognitive functions (learning and memory) by promoting hippocampal neurogenesis and strengthening synaptic plasticity [70]. In healthy individuals, BDNF stimulates VEGF expression through activation of the TrkB receptor and signaling pathways (such as Akt/mTOR). In turn, VEGF, by improving vascularization and microcirculation, facilitates the transport of BDNF and activates progenitor cells to produce new BDNF. However, in patients with TRS, a significant decrease in BDNF impairs VEGF signaling, leading to microangiopathy and reducing synaptic plasticity. Furthermore, chronic inflammation associated with elevated IL-6 and IL-1 causes VEGF to increase BBB permeability instead of stimulating angiogenesis. Peripheral cytokines penetrate the leaky BBB, further suppressing BDNF gene expression, resulting in a decrease in BDNF synthesis [70,71,72]. Studies have shown that BDNF and GDNF jointly support the survival and function of dopaminergic neurons, the activity of which is impaired in schizophrenia. GDNF exerts a strong protective effect on cell bodies, whilst BDNF stimulates the growth of their axons and the release of dopamine [72,73]. A decrease in GDNF concentration has been observed in both serum [74] and CSF [75] in patients with schizophrenia. The main producers of GDNF are astrocytes, and their function may be impaired by the chronic neuroinflammation observed in patients with TRS. Astrocyte dysfunction may lead to a reduction in GDNF production and its neuroprotective effects. In the absence of GDNF, neurons lose their protection against glutamate-induced cytotoxicity. A decrease in GDNF production, combined with reduced BDNF levels, leads to impaired synaptic regeneration, which clinically correlates with the persistence of negative symptoms (anhedonia, social withdrawal) and profound cognitive deficits in TRS [76]. A decrease in BDNF concentration may also be associated with a decrease in NGF synthesis in the brain and reduced peripheral NGF levels, as both have been observed in patients with schizophrenia [77,78]. NGF plays a major role in the proliferation and survival of neurons within the CNS but is specifically responsible for the development and survival of cholinergic neurons in the forebrain [77,79]. The cholinergic system closely regulates synaptic plasticity in the cerebral cortex by stimulating BDNF secretion in glutamatergic neurons. In patients with TRS, marked NGF decrease impairs cholinergic transmission, and this deprives glutamatergic neurons of the stimulus to produce BDNF. As a result, a decrease in BDNF occurs [79]. NT-3 is another neurotrophin that regulates the growth, survival, and plasticity of dopaminergic and cholinergic neurons. Whilst only a few studies to date have shown an increase in serum NT-3 concentrations in chronically ill, treated patients with schizophrenia [20], its potential impact on BDNF concentrations requires further research. Therefore, the decreased BDNF concentration possibly results from multifactorial origins.

Li et al. (2024) [80] reported a statistically significant decrease in the serum MMP-9 after at least 8 weeks of antipsychotic treatment, and this correlated with a favorable clinical response. Conversely, Dickerson et al. (2023) [81] observed that any medication used in chronic SCZ treatment (antipsychotics, other mood stabilizers, antidepressants, and anticholinergic drugs), except for valproic acid, did not influence serum MMP-9 concentration. Additionally, Niitsu et al. (2014) [65] proposed that MMP-9 concentration may be associated with older age and smoking. Significant serum MMP-9 increase was observed in male smokers with chronic SCZ compared to non-smoking males [65]. Since many factors may shape MMP-9 reduction, the precise determination of the reasons responsible for the significant decrease of this protein in the serum of our patients is not possible.

This study also revealed non-significantly decreased serum concentrations of all inhibitors, including TIMP-1, TIMP-2, and TIMP-3, and non-significantly increased concentrations of MMP-7 and MMP-14 in TRS patients (Table 1a). Interpretation of the results seems to be a challenge, as currently, there is no research in which serum levels of metalloproteinases, including MMP-7 and MMP-14, in patients with schizophrenia were measured. Consequently, the positive correlation between MMP-14 and MMP-9 (r = 0.875, BF = 14.366; 95% CI = 0.424 to 0.971) (Table 2 and Table S1) found in our TRS patients before ECT cannot be explained.

It has been suggested that there are relationships between the severity of SCZ symptoms, medical treatment, activation of MMPs, inflammation, and oxidative stress [1,12,24,26,82]. Previous studies have reported that imbalanced OS, expressed as an increased serum concentration of oxidation parameters [reactive oxygen species (ROS), hydrogen peroxide (H_2_O_2_), superoxide dismutase (SOD), malondialdehyde (MDA)] and decreased production of antioxidants, is associated with the severity of clinical symptoms of SCZ [83,84]. Production of OS biomarkers also aggravates the peripheral inflammatory response (IR) [25,85,86]. IR is associated with significantly increased production of pro-inflammatory cytokines [(interleukins: IL-1β, IL-2, IL-6, IL-12), tumor necrosis factor-alpha (TNF-α), and interferon-gamma (IFN-γ)]. The inflammation is counterbalanced by activation of anti-inflammatory factors production [(interleukins: IL-4, IL-5, IL-10), TGF-1β] and neurotrophic defenses (BDNF and antioxidants). The anti-inflammatory response is relatively inadequate to inhibit the inflammatory state and therefore is associated with the severity of SCZ symptoms and poor clinical outcomes in treatment-resistant patients [87,88,89,90,91]. Production of cytokines such as IL-1β and TNF-α may also be facilitated by MMP-9 [92]. Chronic antipsychotic treatment may produce remission of SCZ symptoms through fixing a new homeostatic set-point between inflammatory and anti-inflammatory states [87,90,93]; however, it may increase production of free radicals or decrease production of antioxidants, disrupting the balance between oxidation and antioxidation [34]. The interaction between serum molecules described above presents the hypothetical model, whose confirmation requires further research.

In line with other findings [1,19,41,42], serum BDNF, MMP-9, TIMP-1, and TIMP-2 concentrations in our TRS patients were not correlated with any PANSS scores, i.e., positive, negative, and general symptoms, or the total PANSS score (Table 3 and Table S2). Even if other psychiatric scales, such as the Brief Psychiatric Rating Scale (BPRS) and Clinical Global Impression Improvement (CGI-I), were used, no correlation was consistently found [18,27,40]. However, the baseline concentration of MMP-9 was negatively correlated with amelioration of SCZ symptoms, reflected in the total PANSS score in poor responders to treatment [80]. The only positive correlation in our study was the association between MMP-7 and positive PANSS score (BF = 1.76; 95% CI = 0.038 to 0.915), (Table 3 and Table S2). This suggests that exacerbation of positive symptoms of schizophrenia may be to some extent related to increased serum MMP-7. Concerning MMP-14 and TIMP-3, no correlation with PANSS scales was found in this study, but the interpretation of these findings is difficult due to the lack of data from studies in TRS patients or patients with chronic SCZ.

There are only a few studies on possible correlations between PANSS scores and serum BDNF, MMPs, and their inhibitors in patients with chronic SCZ, and these studies have yielded inconsistent findings. Xiu et al. (2009) [14] reported a positive correlation between decreased levels of BDNF and the PANSS positive score and the total PANSS score. On the contrary, Buckley et al. (2007) [21] found such a correlation only for positive symptoms, whereas Zhang et al. (2007) [22] and Rizos et al. (2008) [94] reported a correlation only for negative symptoms. Other studies found a lack of correlation between BDNF and all PANSS scores, and also no correlation with cognitive decline [61,95]. Regarding MMP-9, increased serum concentration of this MMP was related to poor antipsychotic drug efficacy and exacerbation of SCZ symptoms [80]. The heterogeneity in the association of neurotrophins, MMPs, and their inhibitors concentration with symptoms measured by the PANSS scales may represent the heterogeneity of SCZ itself, the influence of the medication used, and the duration of the illness. Additionally, immunological factors, both pro- and anti-inflammatory, and OS may have a substantial impact.

Further analysis of our data revealed no difference in the serum concentration of BDNF, MMP-7, MMP-9, MMP-14, TIMP-1, TIMP-2, and TIMP-3 post-ECT versus pre-ECT (95% confidence interval contains 0) (Table 4a,c). Concerning BDNF, our results are consistent with findings reported previously [42,43,48,49,96], but in contrast to data from other research, in which increased BDNF after ECT was observed [18,41,47]. It has been suggested that inconsistent results on BDNF changes after ECT may be associated with the sample assay, i.e., whether BDNF was measured in serum (like in our patients) or in plasma [97]. One hypothesis suggests that BDNF is stored first in megakaryocytes and then in platelets, and this is the main mode of peripheral BDNF storage [98]. This BDNF does not cross the blood–brain barrier [99]. As platelets are found in plasma, they are a source of the majority of BDNF measured in serum. However, another theory presumes that BDNF may pass the BBB, becoming the main source of peripherally stored BDNF, but still some BDNF comes from platelets [100]. As a result, BDNF levels measured in plasma after ECT may be higher than in serum, and for this reason, this was observed in some studies [18,41,47]. Nonetheless, other factors such as pharmacological treatment, timepoints of BDNF concentration measurement, or the impact of ECT on BDNF gene expression should be taken into account in future studies. Measuring BDNF levels at different timepoints during ECT therapy and during the follow-up period may contribute to the interpretation of findings. Haghighi et al. (2013) [101] measured serum BDNF levels beginning from the first session of ECT, to 6 months after completion of the therapy in patients with depression. They found a steady increase in serum BDNF level during ECT sessions untill the last follow-up visit, one month after completion of ECT. However, six months later, BDNF concentration was almost the same as before ECT [101]. Whether a similar pattern of serum BDNF changes is possible in TRS patients remains unknown and requires verification.

Our findings on serum TIMP-1 and TIMP-2 are in line with only one published study in TRS patients conducted by Shibasaki et al. (2016) [27]. They also found a decreased level of MMP-9 (*p* = 0.019) post ECT, and this was not confirmed by our study. As the influence of ECT on serum MMP-7, MMP-14, and TIMP-3 in patients with SCZ has not been previously studied, our results may be regarded as unique. We found a weak positive correlation between MMP-14 and TIMP-1 after ECT (BF = 1.44; 95% confidence interval = 0.673 to 0.696) (Table 5a,b). This result indicates that increased serum levels of MMP-14 are associated with increased TIMP-1 concentration in TRS patients.

Further analysis of our data revealed no correlation between serum BDNF, MMP-7, MMP-9, TIMP-1, TIMP-2, TIMP-3, and PANSS scores after ECT (Table 6a,b). Considering MMP-9, TIMP-1, and TIMP-2, our findings are in agreement with Shibasaki et al. (2016), who found no correlation between these parameters and the BPRS scale [27]. However, in the case of BDNF, our results confirm the data of Fernandes et al. (2010) and Akbas et al. (2022) [40,42] but are contrary to other studies [41,49]. Both Li et al. (2016) and Gurjar et al. (2025) [41,49] found a negative correlation between BDNF and PANSS scales. The only credible evidence for positive correlation that was observed in our study was the association between serum MMP-14 and PANSS positive symptoms after ECT (BF = 8.03; 95% confidence interval = 0.337 to 0.965) (Table 6a,b). MMP-14 concentration before ECT did not differ between TRS patients and control subjects, and additionally, it was not correlated with any PANSS score. Post-ECT concentration of MMP-14 did not change significantly as compared to pre-ECT. However, a significant positive correlation was observed between MMP-14 and the intensity of positive symptoms on the PANSS after ECT. Attenuation of productive psychopathology as a result of the ECT may bring TRS patients closer to “normality” and might be associated with the emergence of this correlation.

This study demonstrates that ECT conducted in TRS patients caused a significant decrease in all PANSS scores compared to the PANSS scores pre-ECT. The existence of this difference is confirmed with Bayes Factor and FDR by Benjamini–Hochberg (the details are in Tables S3 and S4). The reduction in the PANSS scores indicates clinical improvement expressed as a reduction in SCZ symptoms.

This result is not unexpected and aligns with previously published studies, in which a combination of ECT with different antipsychotic drugs, including clozapine, was used [18,41,42,43,90]. The effectiveness of such treatment varied from 40 to 71%, regardless of duration of the illness, years of treatment, and number of ECT sessions [45,46,102,103,104]. Additionally, a meta-analysis found higher effectiveness of ECT in clozapine treated patients versus patients treated with other antipsychotics (olanzapine, risperidone, flupenthixol, chlorpromazine, sulpiride) [44]. As the majority of our patients were treated with clozapine, a significant reduction in SCZ symptoms as a result of augmentation with ECT was expected and was beneficial for the patients.

The findings from our study and from other research indicate a decrease in serum BDNF in TRS patients before ECT and a lack of change post-ECT. These findings point to a lack of a clear association between BDNF and clinical response in TRS patients. It has been suggested that ECT may increase the concentration of pro-BDNF, which, by binding to the neurotrophin receptor p75, could lead to cognitive dysfunction in SCZ patients [105]. Cognitive dysfunction post-ECT, in the form of memory problems and disturbances in concentration, was also observed in our patients. It is worth noting that ECT did not increase the serum level of MMP-9, which was evidently decreased in our patients before ECT, and which is responsible for the conversion of pro-BDNF to mature BDNF. The above data and the lack of a correlation between serum BDNF, MMP-9, and PANSS scores post-ECT suggest that BDNF and MMP-9 produced peripherally do not seem to be predictive biomarkers of ECT response. Verification of this hypothesis requires measurement of serum BDNF concentration in a long-term follow-up period after ECT. Moreover, further studies should also include the impact of ECT on other MMPs, including MMP-7, MMP-14, their inhibitors (TIMPs), and their interactions with immune and OS parameters. Otherwise, the interpretation of findings remains largely speculative.

It cannot be forgotten that peripherally produced cytokines, both pro- and anti-inflammatory, may exert their influence on neurotrophins and MMPs in patients with SCZ, and conversely, cytokine concentrations may be modulated by ECT [106]. Previous studies reported a significant decrease in the serum concentration of IL-6, IL-12, IL-10, IL-17, and IP-10 [90,91], no changes in IL-5, TGF-β1, TNF-α, and IL-1β [90,91], or increased concentration in TGF-β1, and IL-4 [107] following ECT in TRS patients. Therefore, a new balance between pro- and anti-inflammatory systems induced by ECT may also modulate changes in neurotrophins, MMPs, and their inhibitors. Particular correlations between cytokines, MMPs such as MMP-7, MMP-9, and MMP-14, and their inhibitors have not been studied so far in TRS patients treated only with antipsychotics, or in combination with ECT.

When interpreting the results of this study, it should also be borne in mind that not only medication, but also the patient’s age, disease duration, type of schizophrenia, and ultra-resistance may be potential confounding factors affecting immune balance. Considering the potential impact of medications used by our patients on biomarkers, data from human studies indicate a generally anti-inflammatory effect of antipsychotics, especially second-generation, in patients with schizophrenia, with evidence of reductions in IL-1β and IFN-γ, and trends toward reductions in IL-6 and TNF-α [108,109]. Nevertheless, many issues remain unclear in TRS patients, i.e., inconsistent patterns of inflammatory markers across studies, substantial differences in methodological approaches, or the clinical relevance of these effects. Moreover, antipsychotic-induced metabolic alterations may also mitigate these effects. Partial alleviation of refractory symptoms by antipsychotics via anti-inflammatory actions, such as reducing peripheral and central inflammatory markers, modulating microglial activation, or influencing immune signaling pathways, remains hypothetical since the evidence is inconsistent and not yet conclusive [110]. For example, clozapine induces inflammatory activation (i.e., increases soluble immune receptors) during initiation in some patients, while in other patients it may induce longer-term normalization of selected immune markers [109,111,112]. Antipsychotic treatment in TRS patients was associated with increased IL-6 and sIL-2R, suggesting that TRS/clozapine populations may not show the anti-inflammatory effect seen in first-episode or acute non-resistant schizophrenia [109]. Reviews describe effects of clozapine on both innate and adaptive immunity, i.e., on T-cell differentiation, Th1/Th2 balance, macrophage responses, microglial activation, cytokines, and chemokines. Clozapine may suppress Th1 differentiation and IFN-γ production; however, clinically, this is counterbalanced by early inflammatory reactions and longer-term inflammation induced by metabolic syndrome [111].

Concerning age, in treatment-resistant schizophrenia (TRS), it is likely to amplify immune vulnerability through immunosenescence/inflammaging, higher medical comorbidity, and altered clozapine pharmacokinetics—but direct age-specific TRS immune data are limited. Most TRS immune studies focus on cytokine profiles rather than age-stratified immune trajectories. TRS is already associated with a pro-inflammatory profile, including higher IL-6, IL-8, TNF-α, sCD25, altered monocyte markers, and dendritic-cell changes [108,113]. With aging, this may overlap with general inflammaging—a chronic low-grade inflammatory state—so older TRS patients may have an additive inflammatory burden from both schizophrenia biology and aging biology. What is more, elevated inflammatory markers may not purely reflect treatment resistance, but in older patients, they may also reflect age-related inflammation, obesity/metabolic syndrome, infection, cardiovascular disease, smoking status, and medication burden [108]. Age also affects clozapine metabolism: older patients tend to have higher clozapine concentrations due to reduced clearance, and guidelines recommend slower titration/lower starting doses in older patients. This matters immunologically because clozapine-related inflammation, infection, pneumonia, myocarditis-like presentations, and neutropenia monitoring become more clinically consequential when age-related frailty and comorbidities are present [108,114].

Other potentially confounding factors influencing the immune system in our TRS patients might be different disease duration, type of schizophrenia, presence of ultra-treatment-resistance, history of substance use, and marked differences in chlorpromazine-equivalent doses of antipsychotics. However, to our best knowledge, no data have been published on the effect of these factors on BDNF concentrations, inflammatory signaling pathways, or MMP/TIMP expression. Our study was performed before the COVID-19 pandemic, and therefore, another possibly important factor influencing the immune system did not exist [115].

It should be emphasized that the results of this preliminary study concern measurements of serum concentrations of BDNF, metalloproteinases, and their inhibitors, as well as ECT’s impact on them. It is worth noting that serum biomarkers are only indirect proxies, and validation using measurements in CSF or neuroimaging is necessary to determine the potential role of BDNF, metalloproteinases, and their inhibitors in schizophrenia [116,117]. The effect of ECT on the concentrations of these biomarkers in CSF also remains an unexplored issue. The results of two available studies showed reduced levels of BDNF pro-peptide [118] and elevated levels of MMP-2 [36] in CSF of patients with schizophrenia. Despite the use of numerous analytical methods, the measurement of mature BDNF in CSF is still impossible due to its low concentration [36]. In contrast, elevated MMP-2 levels were positively correlated with MMP-7 and MMP-10 levels, and, as it was suggested by the authors, it may be associated with the inflammatory process in the brain [36]. Verification of these assumptions requires studies involving a large population of patients with schizophrenia.

## 5. Conclusions

This preliminary study showed no evident differences in serum concentrations of BDNF, metalloproteinases, and their inhibitors in TRS patients pre-ECT compared to healthy controls, except for MMP-9. Furthermore, convincing evidence for a correlation between MMP-9 and MMP-14 in TRS patients was identified. Whether the peripheral changes in BDNF, metalloproteinases, and their inhibitors play any role in TRS development requires verification. Therefore, measurements of the concentrations of these biomarkers in both CSF and serum in larger, better-controlled cohorts are recommended by the authors.

ECT therapy caused marked clinical improvement of schizophrenia symptoms measured by the PANSS, but did not result in evident changes in the serum biomarker concentrations. This finding indicates that the effectiveness of ECT may not be related to peripheral changes in the studied biomarkers, but this statement needs further verification in longitudinal studies including a large number of TRS patients.

## 6. Limitations

We acknowledge some limitations of the current study while interpreting our data. The main disadvantage is a small, heterogeneous group of patients who have been treated with different combinations of antipsychotic drugs (both typical and atypical), mood stabilizers, and antidepressant drugs. All medications may directly or indirectly exert their impact on the concentration of the measured parameters and change the balance between BDNF, MMPs, and their inhibitors. Moreover, treatment with medication was sustained (mood stabilizers were withdrawn, and the doses of the other medications were decreased) during ECT sessions. To determine to what extent ECT solely influences clinical improvement in TRS patients, a sham-controlled study (without concomitant medication) would be ideal, but due to ethical issues combined with withdrawal of the drugs, this is impossible to conduct. It is unclear whether serum concentrations of BDNF, MMPs, and their inhibitors reflect those in the brain, as some reports found no correlation between serum and cerebrospinal fluid (CSF) levels of MMPs [119]. Verification of this matter requires further studies. Last but not least, our sample size was smaller than in other research, and this prevents us from further analysis of factors such as sex, age, duration of the illness, and smoking in relation to the obtained findings.

## Figures and Tables

**Table 1:** (**a**) Descriptive statistics of protein concentration values for pre-ECT patients and the control group. (**b**) Bayes factor independent samples test for pre-ECT vs. controls with Benjamini–Hochberg FDR correction. (**c**) Bayes factor independent samples test for pre-ECT versus controls with Benjamini–Hochberg FDR correction. Posterior distribution with 95% confidence intervals.

(**a**)							
**Protein**	**Pre-ECT (*n* = 8)**			**Control (*n* = 13)**			
	**Mean**	**SD**	**SE-M**	**Mean**		**SD**	**SE-M**
BDNF	90.12	18.37	6.5	94.65		33.16	9.2
MMP-7	1.284	0.731	0.258	0.794		0.613	0.170
MMP-9	68.208	44.287	15.658	255.696		123.394	34.223
MMP-14	1.577	0.450	0.159	1.470		0.352	0.098
TIMP-1	69.975	20.036	7.084	75.575		30.017	8.325
TIMP-2	20.406	7.427	2.626	23.948		7.624	2.114
TIMP-3	0.349	0.138	0.049	0.525		0.582	0.162
(**b**)							
	**Mean Difference**	**Pooled Std. Error Difference**	**Bayes Factor**	**t**	**df**	**Sig. (2-Tailed)**	**BH Corrected *p***
BDNF_l	−0.004	0.061	3.221	−0.063	19	0.950	0.950
MMP-7_l	0.231	0.118	0.765	1.954	19	0.066	0.231
MMP-9_l	−0.607	0.108	0.001	−5.624	19	<0.0001 *	0.0007 *
MMP-14_l	0.026	0.050	2.909	0.505	19	0.619	0.867
TIMP-1_l	−0.013	0.082	3.196	−0.153	19	0.880	0.950
TIMP-2_l	−0.076	0.068	1.954	−1.123	19	0.275	0.642
TIMP-3_l	−0.087	0.110	2.503	0.794	19	0.437	0.765
(**c**)							
		**Posterior**				**95% Confidence Interval**	
		**Mode**	**Mean**	**Variance**		**Lower Bound**	**Upper Bound**
	BDNF_1	−0.004	−0.004	0.004		−0.125	0.118
	MMP-7_l	0.231	0.231	0.018		−0.036	0.497
	MMP-9_l	−0.607	−0.607	0.018		−0.878	−0.337
	MMP-14_l	0.026	0.026	0.004		−0.098	0.149
	TIMP-1_l	−0.013	−0.013	0.007		−0.181	0.156
	TIMP-2_l	−0.076	−0.076	0.007		−0.240	0.088
	TIMP-3_l	−0.087	−0.087	0.012		−0.303	0.129

SD—standard deviation; SE-M—standard error of the mean. *—significance.

**Table 2:** Pairwise correlations between BDNF and MMPs and inhibitor concentrations pre-ECT in TRS patients. Bayes factor inference.

		BDNF Pre	MMP-7 Pre	MMP-9 Pre	MMP-14 Pre	TIMP-1 Pre	TIMP-2 Pre	TIMP Pre
BDNFpre	Pearson Correlation	1	0.217	−0.376	−0.374	−0.284	0.242	0.110
	Bayes Factor		3.399	2.516	2.528	3.062	3.284	3.780
MMP-7pre	Pearson Correlation	0.217	1	−0.248	−0.150	0.171	−0.200	0.611
	Bayes Factor	3.399		3.252	3.663	3.590	3.476	1.024
MMP-9pre	Pearson Correlation	−0.376	−0.248	1	0.875	0.063	−0.529	−0.199
	Bayes Factor	2.516	3.252		0.070	3.870	1.514	3.480
MMP-14pre	Pearson Correlation	−0.374	−0.150	0.875	1	−0.255	**−0.747**	−0.440
	Bayes Factor	2.528	3.663	0.007		3.217	0.386	2.098
TIMP-1pre	Pearson Correlation	−0.284	0.171	0.063	−0.255	1	0.012	0.629
	Bayes Factor	3.062	3.590	3.870	3.217		3.914	0.923
TIMP-2pre	Pearson Correlation	0.242	−0.200	−0.529	**−0.747**	0.012	1	0.148
	Bayes Factor	3.284	3.476	1.514	0.386	3.914		3.670
TIMP-3pre	Pearson Correlation	0.110	0.611	−0.199	−0.440	0.629	0.148	1
	Bayes Factor	3.780	1.024	3.480	2.098	0.923	3.679	

Bayes factor: null versus alternative hypothesis.

**Table 3:** Pairwise correlations between BDNF, MMPs, and inhibitor values with PANSS scores pre-ECT in TRS patients. Bayes factor inference.

		BDNF Pre	MMP-7 Pre	MMP-9 Pre	MMP-14 Pre	TIMP-1 Pre	TIMP-2 Pre	TIMP-3 Pre
PANSS positive symptoms Pre-ECT	Pearson Correlation	−0.164	0.701	0.104	0.254	−0.163	−0.333	0.131
	Bayes Factor	3.614	**0.569**	3.792	3.222	3.653	2.779	3.722
PANSS negative symptoms Pre-ECT	Pearson Correlation	−0.158	0.547	0.456	0.373	0.361	−0.259	0.243
	Bayes Factor	3.636	1.403	1.990	2.529	2.609	3.196	3.277
PANSS general psychopathology Pre-ECT	Pearson Correlation	0.109	0.285	0.304	0.386	0.172	−0.702	−0.140
	Bayes Factor	3.780	3.058	2.950	2.448	3.585	0.563	3.695
PANSS total score Pre-ECT	Pearson Correlation	−0.226	0.608	0.378	0.474	0.081	−0.453	0.012
	Bayes Factor	3.361	1.038	2.497	1.873	3.841	2.009	3.914

Bayes factor: null versus alternative hypothesis.

**Table 4:** (**a**) Descriptive statistics for pre-ECT and post-ECT concentration values. (**b**) Bayes factor for related-samples tests pre-ECT vs. post-ECT with Benjamini–Hochberg FDR correction. (**c**) Bayes factor for related-samples test pre-ECT vs. post-ECT with Benjamini–Hochberg FDR correction. Posterior distribution with 95% confidence intervals.

(**a**)							
**Protein**	**Pre-ECT (*n* = 8)**			**Post-ECT (*n* = 8)**			
	**Mean**	**SD**	**SE-M**	**Mean**		**SD**	**SE-M**
BDNF	90.12	18.37	6.50	105.76		29.36	10.38
MMP-7	1.28	0.73	0.26	0.76		0.21	0.08
MMP-9	68.21	44.29	15.66	80.01		57.70	20.40
MMP-14	1.58	0.45	0.16	1.38		0.39	0.14
TIMP-1	69.98	20.04	7.08	56.70		25.96	9.18
TIMP-2	20.41	7.43	2.63	23.60		9.26	3.27
TIMP-3	0.35	0.14	0.05	0.36		0.15	0.05
(**b**)							
	**Mean Difference/SD**	**Std. Error Mean**	**Bayes Factor**	**t**	**df**	**Sig. (2-Tailed)**	**BH** **Corrected *p***
BDNF	−0.063	0.050	1.983	−1.261	7	0.248	0.755
pre/post	SD 0.141						
MMP-7	0.183	0.103	1.146	1.776	7	0.119	0.728
pre/post	SD 0.292						
MMP-9	−0.042	0.136	3.742	−0.309	7	0.766	0.866
pre/post	SD 0.383						
MMP-14	0.058	0.073	2.944	0.791	7	0.455	0.833
pre/post	SD 0.206						
TIMP-1	0.129	0.119	2.327	1.088	7	0.312	0.796
pre/post	SD 0.335						
TIMP-2	−0.06	0.092	3.228	−0.646	7	0.539	0.854
pre/post	SD 0.261						
TIMP-3	−0.013	0.070	3.848	−0.190	7	0.854	0.868
pre/post	SD 0.199						
(**c**)							
		**Posterior**				**95% Confidence Interval**	
**Variable**		**Mode**	**Mean**	**Variance**		**Lower Bound**	**Upper Bound**
BDNFpre_l–BDNFpost_l		−0.063	−0.063	0.006		−0.214	0.089
MMP-7pre_l–MMP7post_l		0.183	0.183	0.025		−0.131	0.497
MMP-9pre_l–MMP-9post_l		−0.042	−0.042	0.043		−0.454	0.371
MMP-14pre_l–MMP-14post_l		0.058	0.058	0.012		−0.164	0.279
TIMP-1pre_l–TIMP-1post_l		0.129	0.129	0.033		−0.232	0.490
TIMP-2pre_l–TIMP-2post_l		−0.060	−0.060	0.020		−0.340	0.221
TIMP-3pre_l–TIMP-3post_l		−0.013	−0.013	0.012		−0.227	0.201

Bayes factor: null versus alternative hypothesis. Prior on variance: diffuse. Prior on mean: diffuse.

**Table 5:** (**a**) Pairwise correlations between BDNF, MMPs, and inhibitor concentrations post-ECT in TRS patients. Bayes factor inference. (**b**) Correlations between BDNF, MMPs, and inhibitor concentrations post-ECT in TRS patients. Posterior distribution characterization with 95% confidence intervals.

(**a**)									
			**BDNF Post**	**MMP-7 Post**	**MMP-9 Post**	**MMP-14 Post**	**TIMP-1 Post**	**TIMP-2 Post**	**TIMP-3 Post**
BNDFpost	Pearson Correlation		1	−0.223	−0.084	−0.365	−0.138	−0.016	−0.086
	Bayes Factor			3.372	3.835	2.584	3.703	3.913	3.831
MMP-7post	Pearson Correlation		−0.223	1	−0.059	−0.123	−0.055	0.335	−0.204
	Bayes Factor		3.372		3.875	3.746	3.881	2.765	3.458
MMP-9post	Pearson Correlation		−0.084	−0.059	1	0.328	0.383	0.239	0.375
	Bayes Factor		3.835	3.875		2.810	2.467	3.300	2.521
MMP-14post	Pearson Correlation		−0.365	−0.123	0.328	1	0.673	−0.648	−0.046
	Bayes Factor		2.584	3.746	2.810		0.696	0.823	3.892
TIMP-1post	Pearson Correlation		−0.138	−0.055	0.383	0.673	1	−0.262	−0.039
	Bayes Factor		3.703	3.881	2.467	0.696		3.184	3.898
TIMP-2post	Pearson Correlation		−0.016	0.335	0.239	−0.648	−0.262	1	0.528
	Bayes Factor		3.913	2.765	3.300	0.823	3.184		1.525
TIMP-3post	Pearson Correlation		−0.086	−0.204	0.375	−0.046	−0.039	0.528	1
	Bayes Factor		3.831	3.458	2.521	3.892	3.898	1.525	
(**b**)									
			**BDNF Post**	**MMP-7 Post**	**MMP-9 Post**	**MMP-14 Post**	**TIMP-1 Post**	**TIMP-2 Post**	**TIMP-3 Post**
BDNF post	Posterior	Mode		−0.346	−0.135	−0.529	−0.219	−0.026	−0.139
		Mean		−0.143	−0.051	−0.240	−0.085	−0.011	−0.054
		Variance		0.091	0.091	0.084	0.092	0.090	0.091
	95% Confidence Interval	Lower Bound		−0.690	−0.618	−0.765	−0.640	−0.586	−0.625
		Upper Bound		0.433	0.522	0.319	0.499	0.543	0.511
MMP-7 post	Posterior	Mode	−0.346		−0.096	−0.196	−0.089	0.494	−0.319
		Mean	−0.143		−0.037	−0.080	−0.028	0.217	−0.128
		Variance	0.091		0.094	0.092	0.091	0.087	0.093
	95% Confidence Interval	Lower Bound	−0.690		−0.616	−0.647	−0.610	−0.343	−0.676
		Upper Bound	0.433		0.541	0.492	0.534	0.756	0.460
MMP-9 post	Posterior	Mode	−0.135	−0.096		0.485	0.550	0.367	0.541
		Mean	−0.051	−0.037		0.219	0.255	0.154	0.243
		Variance	0.091	0.094		0.086	0.085	0.090	0.087
	95% Confidence Interval	Lower Bound	−0.618	−0.616		−0.365	−0.330	−0.427	−0.333
		Upper Bound	0.522	0.541		0.730	0.762	0.693	0.780
MMP-14 post	Posterior	Mode	−0.529	−0.196	0.485		0.815	−0.796	−0.074
		Mean	−0.240	−0.080	0.219		0.498	−0.473	−0.030
		Variance	0.084	0.092	0.086		0.064	0.066	0.092
	95% Confidence Interval	Lower Bound	−0.765	−0.647	−0.365		−0.009	−0.915	−0.585
		Upper Bound	0.319	0.492	0.730		0.908	0.015	0.550
TIMP-1post	Posterior	Mode	−0.219	−0.089	0.550	0.815		−0.399	−0.064
		Mean	−0.085	−0.028	0.255	0.498		−0.169	−0.030
		Variance	0.092	0.091	0.085	0.064		0.090	0.091
	95% Confidence Interval	Lower Bound	−0.640	−0.610	−0.330	−0.009		−0.713	−0.608
		Upper Bound	0.499	0.534	0.762	0.908		0.403	0.537
TIMP-2 post	Posterior	Mode	−0.026	0.494	0.367	−0.796	−0.399		0.697
		Mean	−0.011	0.217	0.154	−0.473	−0.169		0.362
		Variance	0.090	0.087	0.090	0.066	0.090		0.078
	95% Confidence Interval	Lower Bound	−0.586	−0.343	−0.427	−0.915	−0.713		−0.199
		Upper Bound	0.543	0.756	0.693	0.015	0.403		0.830
TIMP-3 post	Posterior	Mode	−0.139	−0.319	0.541	−0.074	−0.064	0.697	
		Mean	−0.054	−0.128	0.243	−0.030	−0.030	0.362	
		Variance	0.091	0.093	0.087	0.092	0.091	0.078	
	95% Confidence Interval	Lower Bound	−0.625	−0.676	−0.333	−0.585	−0.608	−0.199	
		Upper Bound	0.511	0.460	0.780	0.550	0.537	0.830	

Bayes factor: null versus alternative hypothesis. The Jeffreys prior analyses assumed reference priors (c = −1.5).

**Table 6:** (**a**) Pairwise correlations between BDNF, MMPs, and inhibitor values with PANSS scores post-ECT in TRS patients. Bayes factor inference. (**b**) Pairwise correlations between BDNF, MMPs, and inhibitor values and PANSS scores post-ECT in TRS patients. Posterior distribution characterization with 95% confidence intervals.

(**a**)									
			**BDNF Post**	**MMP-7 Post**	**MMP-9 Post**	**MMP-14 Post**	**TIMP-1 Post**	**TIMP-2 Post**	**TIMP-3 Post**
PANSS positive symptoms Post-ECT	Pearson Correlation		−0.698	0.188	0.253	0.842	0.609	−0.309	−0.036
	Bayes Factor		0.583	3.525	3.228	**0.125**	1.033	2.920	3.901
PANSS negative symptoms Post-ECT	Pearson Correlation		0.070	−0.459	0.054	0.042	−0.378	−0.393	0.134
	Bayes Factor		3.860	1.971	3.883	3.896	2.498	2.402	3.713
PANSS general psychopathology Post-ECT	Pearson Correlation		−0.162	0.036	0.217	0.190	−0.254	−0.211	−0.434
	Bayes Factor		3.623	3.900	3.401	3.515	3.223	3.428	2.138
PANSS total score Post-ECT	Pearson Correlation		−0.384	0.009	0.221	0.486	−0.140	−0.465	−0.368
	Bayes Factor		2.464	3.915	3.384	1.796	3.694	1.929	2.565
(**b**)									
			**BDNF Post**	**MMP-7 Post**	**MMP-9 Post**	**MMP-14 Post**	**TIMP-1 Post**	**TIMP-2 Post**	**TIMP-3 Post**
PANSS positive symptoms Post-ECT	Posterior	Mode	−0.832	0.295	0.387	0.921	0.766	−0.461	−0.058
		Mean	−0.527	0.122	0.163	**0.702**	0.438	−0.203	−0.026
		Variance	0.058	0.091	0.090	**0.033**	0.070	0.088	0.091
	95% Confidence Interval	Lower Bound	−0.921	−0.432	−0.434	**0.337**	−0.099	−0.764	−0.581
		Upper Bound	−0.049	0.702	0.698	**0.965**	0.870	0.353	0.557
PANSS negative symptoms Post-ECT	Posterior	Mode	0.113	−0.632	0.087	0.067	−0.545	−0.562	0.214
		Mean	0.040	−0.308	0.033	0.030	−0.249	−0.256	0.087
		Variance	0.092	0.081	0.091	0.091	0.086	0.086	0.091
	95% Confidence Interval	Lower Bound	−0.558	−0.818	−0.545	−0.520	−0.776	−0.779	−0.485
		Upper Bound	0.596	0.240	0.587	0.613	0.325	0.315	0.657
PANSS general psychopathology Post-ECT	Posterior	Mode	−0.256	0.059	0.337	0.299	−0.389	−0.328	−0.605
		Mean	−0.102	0.024	0.136	0.122	−0.164	−0.133	−0.289
		Variance	0.090	0.092	0.092	0.091	0.090	0.090	0.082
	95% Confidence Interval	Lower Bound	−0.659	−0.529	−0.447	−0.461	−0.709	−0.686	−0.800
		Upper Bound	0.464	0.609	0.689	0.682	0.416	0.446	0.260
PANSS total score Post-ECT	Posterior	Mode	−0.551	0.014	0.342	0.658	−0.224	−0.638	−0.533
		Mean	−0.245	0.007	0.138	0.329	−0.092	−0.311	−0.245
		Variance	0.086	0.091	0.090	0.080	0.092	0.082	0.086
	95% Confidence Interval	Lower Bound	−0.766	−0.565	−0.456	−0.203	−0.670	−0.813	−0.758
		Upper Bound	0.318	0.576	0.671	0.848	0.484	0.249	0.339

Bayes factor: null versus alternative hypothesis. The Jeffreys prior analyses assume reference priors (c = −1.5).

## Data Availability

The original contributions presented in this study are included in the article/Supplementary Materials. Further inquiries can be directed to the corresponding author.

## Supplementary Materials

The following supporting information can be downloaded at: https://www.mdpi.com/article/10.3390/biomedicines14071535/s1, Table S1. Correlations between BDNF, MMPs and inhibitor concentrations pre-ECT in TRS patients. Posterior distribution characterization with 95% confidence intervals. Table S2. Pairwise correlations between BDNF, MMPs, and inhibitor values with PANSS pre-ECT in TRS patients. Posterior distribution characterization with 95% Confidence Intervals. Table S3. Bayes factor for related T-Test with FDR correction by Benjamini–Hochberg. Table S4. Posterior Distribution Characterization for Related-Sample Mean Difference.

## Abbreviations

Blood–brain barrier (BBB); brain-derived neurotrophic factor (BDNF); Brief Psychiatric Rating Scale (BPRS); central nervous system (CNS); Clinical Global Impression Improvement (CGI-I); cerebrospinal fluid (CSF); computed tomography (CT); electroconvulsive therapy (ECT); GDNF (glial cell line-derived neurotrophic factor), hydrogen peroxide (H_2_O_2_); IL- (interleukin); Inflammatory response (IR); interferon γ (IFN-γ); long-term potentiation (LTP); NGF (nerve growth factor), NT-3 (neurotrophin-3), malondialdehyde (MDA); metalloproteinases (MMPs); oxidative stress (OS); Positive and Negative Syndrome Scale (PANSS); reactive oxygen species (ROS); schizophrenia (SCZ); superoxide dismutase (SOD); repetitive transcranial magnetic stimulation (rTMS); transcranial direct stimulation (tDCS); tissue inhibitor of metalloproteinase (TIMP); transforming growth factor β1 (TGF-β1); treatment-resistant schizophrenia (TRS); ultra treatment-resistant schizophrenia (UTRS); VEGF (vascular endothelial growth factor); tumor necrosis factor-alpha (TNF-α).
