# Supplementary material for: Neurotrophins and Matrix Metalloproteinases in Treatment-Resistant Schizophrenia: Effects of Electroconvulsive Therapy on Serum Biomarkers and Clinical Outcomes: A Preliminary Study
Source: Biomedicines. 2026 Jul 8;14(7):1535. doi: 10.3390/biomedicines14071535 (PMC13406290; doi:10.3390/biomedicines14071535)
Supplement: Supplementary file 1 [file biomedicines-14-01535-s001.zip › biomedicines-4352898-supplementary.pdf]

Table S1. Correlations between BDNF, MMPs, and inhibitor concentrations pre-ECT in TRS patients. Posterior distribution characterization with 95% confidence intervals.

			BDNF pre	MMP-7pre	MMP-9 pre	MMP-14 pre	TIMP-1pre	TIMP-2pre	TIMP-3pre
BDNFpre	Posterior	Mode		0.338	-0.541	-0.539	-0.429	0.372	0.176
		Mean		0.141	-0.248	-0.247	-0.183	0.149	0.065
		Variance		0.090	0.086	0.086	0.089	0.089	0.094
	95% Confidence	Lower Bound		-0.443	-0.784	-0.786	-0.740	-0.439	-0.520
	Interval	Upper Bound		0.681	0.303	0.309	0.376	0.679	0.637
MMP-7pre	Posterior	Mode	0.338		-0.381	-0.239	0.270	-0.313	0.767
		Mean	0.141		-0.154	-0.102	0.108	-0.122	0.438
		Variance	0.090		0.089	0.090	0.090	0.089	0.071
	95% Confidence	Lower Bound	-0.443		-0.719	-0.678	-0.466	-0.663	-0.091
	Interval	Upper Bound	0.681		0.409	0.454	0.660	0.462	0.889
MMP-9pre	Posterior	Mode	-0.541	-0.381		0.939	0.103	-0.699	-0.311
		Mean	-0.248	-0.154		0.755	0.036	-0.369	-0.126
		Variance	0.086	0.089		0.026	0.091	0.078	0.090
	95% Confidence	Lower Bound	-0.784	-0.719		0.424	-0.534	-0.846	-0.684
	Interval	Upper Bound	0.303	0.409		0.971	0.605	0.184	0.438
MMP-14pre	Posterior	Mode	-0.539	-0.239	0.939		-0.390	-0.865	-0.612
		Mean	-0.247	-0.102	0.755		-0.163	-0.581	-0.299
		Variance	0.086	0.090	0.026		0.089	0.052	0.081
	95% Confidence	Lower Bound	-0.786	-0.678	**0.424**		-0.707	-0.928	-0.790
	Interval	Upper Bound	0.309	0.454	**0.971**		0.413	-0.122	0.269
TIMP-1pre	Posterior	Mode	-0.429	0.270	0.103	-0.390		0.019	0.782
		Mean	-0.183	0.108	0.036	-0.163		0.008	0.457
		Variance	0.089	0.090	0.091	0.089		0.091	0.067
	95% Confidence	Lower Bound	-0.740	-0.466	-0.534	-0.707		-0.553	-0.047
	Interval	Upper Bound	0.376	0.660	0.605	0.413		0.594	0.890
TIMP-2pre	Posterior	Mode	0.372	-0.313	-0.699	-0.865	0.019		0.236
		Mean	0.149	-0.122	-0.369	-0.581	0.008		0,0092
		Variance	0.089	0.089	0.078	0.052	0.091		0.090
	95%	Lower	-0.439	-0.663	-0.846	**-0.928**	-0.553		-0.484

	Confidence	Bound							
	Interval	Upper Bound	0.679	0.462	0.184	**-0.122**	0.594		0.644
TIMP-3pre	Posterior	Mode	0.176	0.767	-0.311	-0.612	0.782	0.236	
		Mean	0.065	0.438	-0.126	0.299	0.457	0.092	
		Variance	0.094	0.071	0.090	0.081	0.067	0.090	
	95% Confidence	Lower Bound	-0.520	-0.091	-0.684	-0.790	-0.047	-0.484	
	Interval	Upper Bound	0.637	0.889	0.438	0.269	0.089	0.644	

The Jeffreys on prior analyses assume reference priors (c=-1,5)

Table S2. Pairwise correlations between BDNF, MMPs, and inhibitor values with PANSS pre-ECT in TRS patients. Posterior distribution characterization with 95% Confidence Intervals.

			BDNF pre	MMP-7 pre	MMP-9 pre	MMP-14 pre	TIMP-1 pre	TIMP-2 pre	TIMP-3 pre
PANSS positive symptoms Pre-ECT	Posterior	Mode	-0.260	0.834	0.168	0.389	-0.243	-0.491	0.209
		Mean	-0.106	0.525	0.067	0.156	-0,100	-0.217	0.079
		Variance	0.090	0.059	0.092	0.090	0,090	0,088	0.093
	95% Confidence Interval	Lower Bound	-0.666	**0.038**	-0.488	-0.425	-0.656	-0.759	-0.506
		Upper Bound	0.472	**0.915**	0.654	0.699	0.470	0.356	0.651
PANSS negative symptoms Pre-ECT	Posterior	Mode	-0.251	0.714	0.629	0.539	0.524	-0.396	0.374
		Mean	-0.104	0.382	0.311	0.242	0.233	-0.166	0.152
		Variance	0.091	0.074	0.080	0.086	0.088	0.091	0.090
	95% Confidence Interval	Lower Bound	-0.665	-0.152	-0.268	-0.336	-0.329	-0.700	-0.447
		Upper Bound	0.472	0.845	0.783	0.758	0.785	0.429	0.686
PANSS general psychopathology Pre-ECT	Posterior	Mode	0.175	0.430	0.455	0.553	0.272	-0.835	-0.223
		Mean	0.062	0.185	0.193	0.251	0.107	-0.527	-0.089
		Variance	0.094	0.090	0.090	0.086	0.091	0.060	0.092
	95% Confidence Interval	Lower Bound	-0.500	-0.390	-0.386	-0,311	-0.450	-0.926	-0.634
		Upper Bound	0.651	0.737	0.731	0.782	0.678	-0.050	0.507
PANSS total score Pre-ECT	Posterior	Mode	-0.350	0.765	0.545	0.646	0.130	-0.626	0.020
		Mean	-0.143	0.438	0.246	0.318	0.049	-0.305	0.003
		Variance	0.090	0.070	0.086	0.082	0.090	0.082	0.092
	95% Confidence Interval	Lower Bound	-0.690	-0.081	-0.330	-0.250	-0.498	-0.800	-0.577
		Upper Bound	0.436	0.879	0.754	0.808	0.638	0.253	0.568

The Jeffreys on prior analyses assume reference priors (c=-1,5)

Table S3. Bayes factor for related T-Test with FDR correction by Benjamini–Hochberg.

	Mean Difference	Standard Deviation	Standard Error Mean	Bayes Factor	t	df	Sig.(2-tailed)	BH corrected p	Bayes Factor HA v H0
PANSS Positive symptoms Pre – PANSS Positive symptoms Post	0.216	0.130	0.046	0.041	4.697	7	0,00222	0.002	24,511*
PANSS Negative symptoms Pre- PANSS Negative symptoms Post	0.166	0.093	0.033	0.028	5.063	7	0,00146	0,0013	35,1848*
PANSS General Psychopathology Pre - PANSS General Psychopathology Post	0.183	0.075	0.027	0.006	6.874	7	0,00020	0.001	167,4441 **
PANSS Total score Pre - PANSS Total score Post	0.183	0.056	0.020	0.001	9.230	7	0,00004	0.005	838,4481 **

Bayes factor: Null versus alternative hypothesis

*- very strong support for alternative hypothesis

**- extremely strong support for alternative hypothesis

Table S4. Posterior Distribution Characterization for Related-Sample Mean Difference.

	**Posterior Mode**	**Posterior Mean**	**Posterior Variance**	**95% Credible Intervals Lower Bound**	**95% Credible Intervals Upper Bound**
PANSS Positive symptoms Pre – PANSS Positive symptoms Post	0.216	0.216	0.005	0.076	0.356
PANSS Negative symptoms Pre- PANSS Negative symptoms Post	0.166	0.166	0.003	0.066	0.266
PANSS General Psychopathology Pre - PANSS General Psychopathology Post	0.183	0.183	0.002	0.102	0.264
PANSS Total score Pre - PANSS Total score Post	0.183	0.183	0.001	0.123	0.244

Prior on Variance: Diffuse. Prior on Mean: Diffuse.
